# Association of Guillain-Barre Syndrome With COVID-19: A Case Report and Literature Review

**DOI:** 10.7759/cureus.13828

**Published:** 2021-03-11

**Authors:** Romil Singh, Saher T Shiza, Rabeea Saadat, Manal Dawe, Usama Rehman

**Affiliations:** 1 Critical Care, Mayo Clinic, Rochester, USA; 2 Internal Medicine, Deccan College of Medical Sciences, Hyderabad, IND; 3 Pediatrics, Holy Family Hospital, Rawalpindi, PAK; 4 Medicine, Capital Medical University, Beijing, CHN; 5 Anaesthesia, Mayo Hospital, Lahore, PAK

**Keywords:** guillain-barre syndrome, covid-19, sars-cov-2, amasn, acute motor and sensory axonal neuropathy

## Abstract

There is growing evidence of neurological involvement in patients with coronavirus disease 19 (COVID-19), suggesting that Guillain-Barre syndrome (GBS) can also occur with severe acute respiratory syndrome coronavirus 2 (SARS-CoV-2) as a neurological complication. Herein, we describe a unique case of a 45-year-old male who manifested with sudden onset progressive symmetric ascending paralysis leading to quadriplegia one week after developing fever, cough, and dyspnea. On examination, he had areflexia in lower limbs and hyporeflexia in upper limbs. Hypoesthesia to fine touch and vibration distal to calf was noted. His reverse transcriptase-polymerase chain reaction (RT-PCR) was positive for COVID-19, and his cerebrospinal fluid (CSF) analysis revealed albumin-cytologic dissociation. The diagnosis of GBS was made based on clinical presentation and neurophysiological studies due to COVID-19. He was commenced on intravenous immunoglobulin, and improvement in his condition was observed. He was discharged to a rehabilitation center for regular physical therapy.

## Introduction

Severe acute respiratory syndrome coronavirus 2 (SARS-CoV-2), the causative agent of coronavirus disease 19 (COVID-19), generally presents as a respiratory infection. The severity of symptoms may vary from a common cold-like illness to severe lung infection leading to acute respiratory distress syndrome (ARDS), which is highly fatal. The infection generally presents with typical symptoms, such as fever, dyspnea, and cough, although some patients may be asymptomatic [1-2]. Complications of severe infection, including septic shock, embolism, and multi-organ failure, have been reported in old age and patients with comorbidities. As the number of affected patients has grown, reports of neurological manifestations are increasing, which include altered mental status, Guillain-Barre syndrome (GBS), encephalopathy, psychosis, neurocognitive (dementia-like) syndrome, ischemic strokes, intracerebral hemorrhage, cerebral nervous system (CNS) vasculitis, and other cerebrovascular accidents. The literature has also underlined a correlation between GBS and a previous COVID-19 infection [3]. That correlation can either be considered a direct effect of the virus on the nervous system, para-infectious complication, or post-infectious immune-mediated response. Here, we describe a patient with an acute motor and sensory axonal neuropathy (AMSAN) variant of GBS following COVID-19.

## Case presentation

A 45-year-old male with a past medical history of hypothyroidism was brought to the emergency department with sudden-onset, progressive, symmetric ascending weakness followed by worsening weakness and quadriplegia the night before admission. He had a history of cough, fever, and intermittent dyspnea one week before the onset of neurological manifestations. He had no history of fall, travel, or trauma. He had no bowel or urinary incontinence.

On examination, he had a temperature of 99^o^F, blood pressure of 125/85 mmHg, heart rate of 85 beats per minute, respiratory rate of 21 per minute, and oxygen saturation of 96% at room air. He was well-oriented in time, place, and person. After consulting with an infectious disease specialist, he underwent the testing of COVID-19. His reverse transcriptase-polymerase chain reaction (RT-PCR) came positive, and he was started on remdesivir and azithromycin. He had bilateral facial paresis with no signs of meningeal irritation or upper motor neuron lesion. Neurological examination revealed decreased muscle strength in all the limbs with a Medical Research Council (MRC) score of 2/5 in the proximal and 4/5 in the distal part of the upper limbs, and 1/5 in the distal and 2/5 in the proximal parts of the lower limbs. The patient had areflexia in lower limbs and hyporeflexia in upper limbs with no evidence of ataxia. He also complained of numbness and tingling over the lower extremities. On examination, hypoesthesia to fine touch and vibration distal to calf was noted. His initial blood investigations are shown in Table 1. The cardiovascular and respiratory system review was normal.

**Table 1:** Results of initial blood workup WBC; white blood cell, RBC; red blood cell, AST; aspartate aminotransferase, ALT; alanine aminotransferase, CRP; C-reactive protein, ESR; erythrocyte sedimentation rate

Blood workup	Results	Reference value	Unit
WBC	13.8	4.0-11.0	x E9/L
RBC	4.3	4.0-5.1	x E12/L
Neutrophil	7.9	2.0-7.5	x E9/L
Lymphocytes	3.1	1.0-3.5	x E9/L
Hemoglobin	12.9	14-17	g/dL
Platelet count	239	150-400	x E9/L
Sodium	139	136-145	mmol/L
Chloride	101	98-106	mmol/L
Potassium	3.9	3.5-5.0	mmol/L
Urea nitrogen	14	8.0-20	mg/dL
Creatinine	0.9	0.7-1.2	mg/dL
Blood glucose	145	< 200	mg/dL
AST	41	8-48	IU/L
ALT	33	7-55	IU/L
CRP	10	0.8-100	mg/L
ESR	68	< 22	

**Figure 1:**
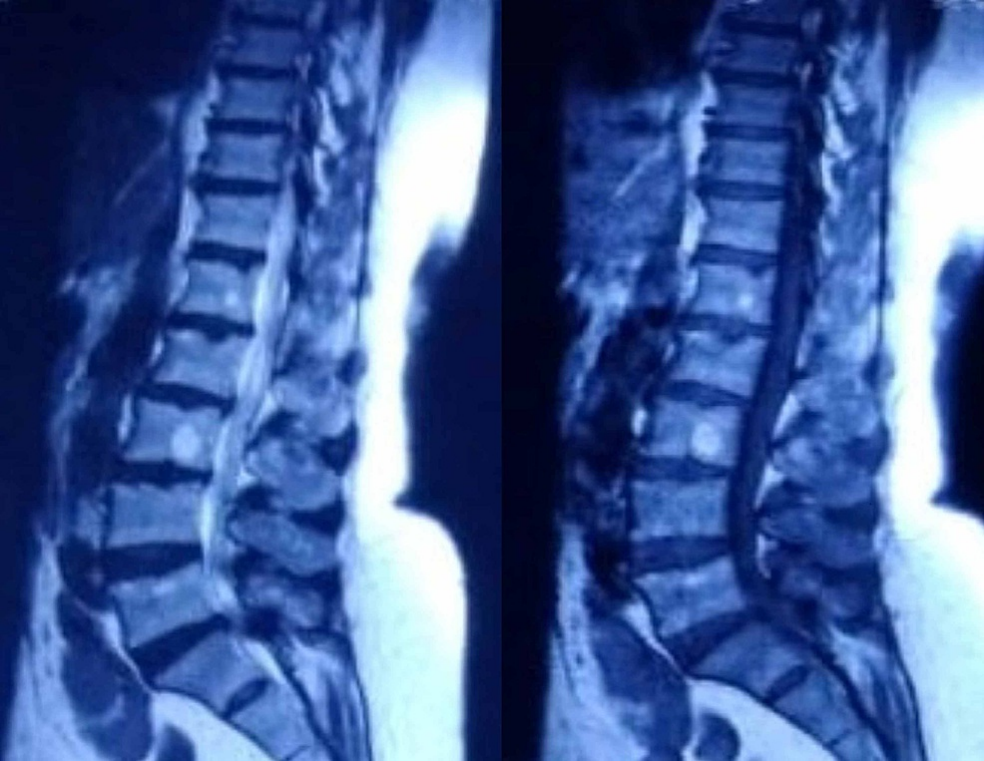
MRI of the lumbosacral spine without any significant findings MRI, magnetic resonance imaging

Cerebrospinal fluid (CSF) analysis on his second day of admission revealed albumin-cytologic disassociation (protein > 1.27 mg/L), with normal cell count and absence of oligoclonal bands. Magnetic resonance imaging (MRI) of the spine was unremarkable, with old age changes (Figure 1).

**Figure 2:**
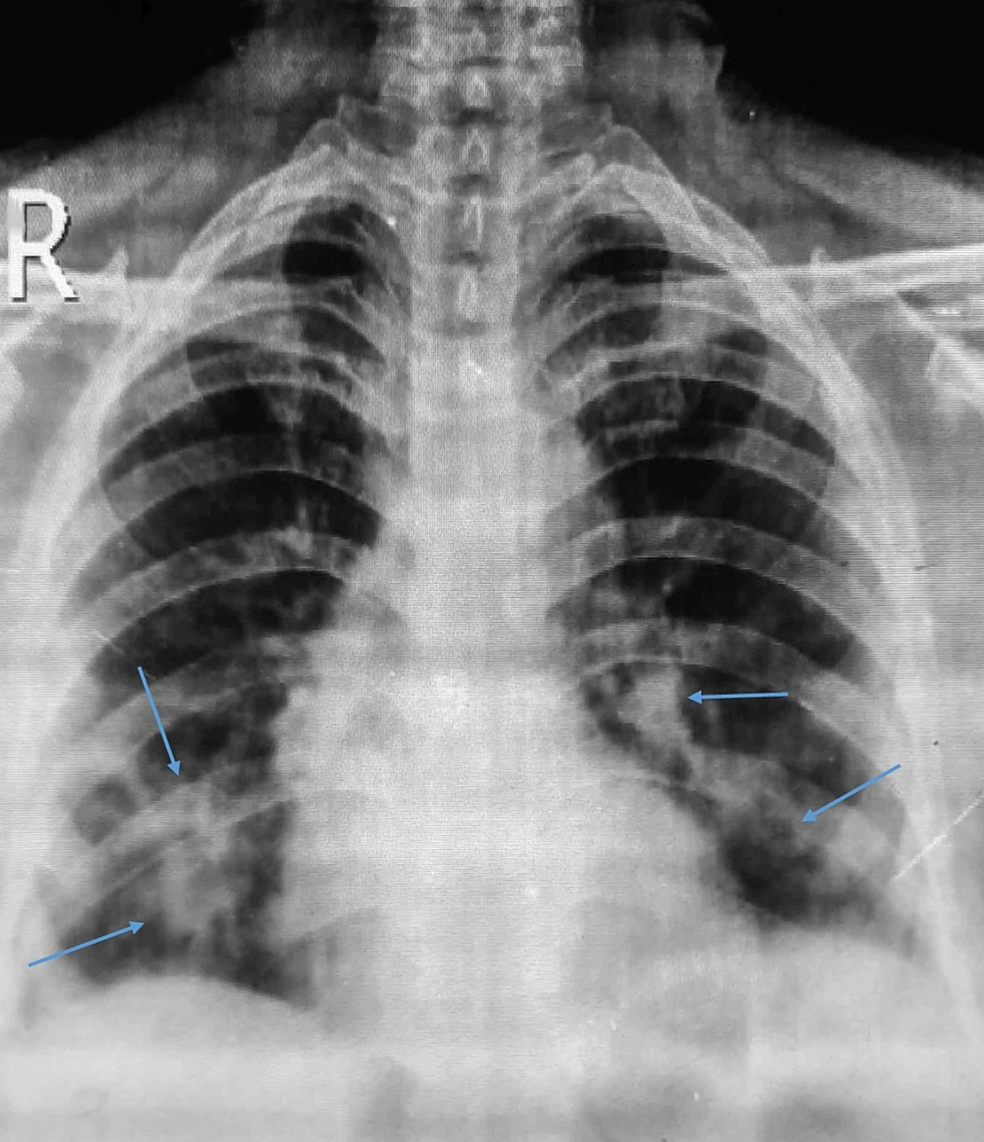
Chest X-ray showing scattered infiltrates in both lungs (blue arrows)

Chest X-ray revealed scattered infiltrates in the lower lobe of both lungs (Figure 2). The neurophysiological study demonstrated decreased amplitude, normal distal latencies, and prolonged F-wave latencies. Electromyogram showed decreased recruitment (Table 2).

**Table 2:** Electromyogram showing decreased response in both sensory and motor nerves NR; no response, ms; millisecond

Nerve stimulated	Stimulation site	Amplitude (motor=mV, sensory=µV)		Latency (ms)		Conduction velocity		F wave (ms)	
		Left	Right	Left	Right	Left	Right	Left	Right
Tibial (motor)	Ankle	0.4	1.00	12.09	14.11			21	29
	Popliteal fossa	0.4	0.90	27.4	26.21	23	27		
Peroneal (motor)	Ankle	NE	NE	NE	NE	NE	NE	NE	NE
Median (motor)	Wrist	1.8	2.3	7.59	6.61			49	52
	Antecubital fossa	1.3	1.9	14.27	13.90	41	31		
Ulnar (motor)	Wrist	4.4	4.7	4.69	4.17			47	50
	Below elbow	3.5	4.6	8.52	8.22				
	Above elbow	3.4	4.5	10.20	9.90	39	46		
Median (sensory)	Wrist	NE	NE	NE	NE	NE	NE		
Ulnar (sensory)	Wrist	NE	NE	NE	NE	NE	NE		
Sural (sensory)	Calf	4.5	NE	2.0	NE	35	NE		

A diagnosis of GBS due to COVID-19 was made, and the findings were consistent with the AMASN variant of GBS. He was started on 0.40 g/kg per day intravenous immunoglobulin (IVIG) for five days in addition to drugs initiated for COVID-19 (remdesivir, azithromycin). His respiratory status was monitored periodically, along with the vitals. The patient's condition improved significantly on the following day, and he started regaining his muscle strength back. Power of the muscles and limb sensation improved significantly. He was discharged to a rehabilitation center for regular physical therapy.

## Discussion

SARS-CoV-2 is one of the seven types of coronaviruses that cause severe lung infection such as middle east respiratory syndrome (MERS) and SARS. Along with various common symptoms like fever, cough, dyspnea, body aches, and nausea, manifestations like pneumonia, liver diseases, respiratory failure, cardiac issues, stroke, and other neurological complications have been reported. One proposed mechanism of action is that SARS-CoV-2 distinguishes the angiotensin-converting enzyme 2 (ACE2) receptors while accessing the host cell receptor. High expression of the ACE2 receptors has been observed in the lung, gastrointestinal tract, cardiomyocytes, urothelial cells, and proximal tubular cells, affecting these systems. The neurons and glial cells of the nervous system also have an expression of ACE2 receptors, making the brain a potential target of SARS-CoV-2 presenting in the form of stroke, encephalopathies, vasculitis, and GBS [4].

GBS is an immune-mediated disorder related to the peripheral nervous system. Progressive ascending weakness of the limbs and reduction in or loss of reflexes is the typical GBS presentation. On CSF analysis in this disease, the protein concentrations increase; however, the white cell count remains normal. A viral or bacterial infection is usually a causative agent of GBS. The immune system is triggered in response to the antigen. The nerve roots and peripheral nerves are inflamed and injured due to the structural similarity of this antigen to axons and myelin sheath. The symptoms peak within four weeks and warrant careful patient monitoring because 20% to 30% of them will require mechanical ventilation [5].

GBS is usually seen after infection by campylobacter jejune, cytomegalovirus, Zika virus, and the Epstein-Barr virus. The increasing evidence of GBS after a preceding COVID-19 attack seems another addition to the list and raises concerns about the neurological and immune-mediated complications of COVID-19. To our knowledge, 29 articles reported 33 cases of GBS associated with SARS COV-2 in addition to our reported case [6]. AMSAN is an uncommon variant of GBS reported in COVID-19 infection, and we have underlined cases of AMSAN variant of GBS in COVID-19 disease so far (Table 3).

**Table 3:** COVID-19 cases-associated AMSAN variant of Guillain-Barre syndrome EMG, electromyography; CSF, cerebrospinal fluid; MRI, magnetic resonance imaging; AMSAN, acute motor and sensory axonal neuropathy; ACD, albumin-cytologic disassociation; IVIG, intravenous immunoglobulin; M, male; F, female; MRI, magnetic resonance imaging; COVID-19, coronavirus disease 19

Author	Age (yrs)	Sex	Time between events (days)	Clinical features	EMG	CSF analysis	MRI	Treatment
Toscano et al. [7]	77	F	07	Tetraplegia, areflexia, paranesthesia in upper limbs, facial diplopia, dysphagia, tongue weakness, respiratory failure	AMSAN	ACD	spine: enhancement of caudal never roots	Two cycles of IVIG,s poor outcomes
Toscano et al. [7]	23	M	Zero	Facial paresis, areflexia, lower limbs paranesthesia, ataxia	AMSAN	ACD	Brain: enhancement of facial never bilaterally	IVIG, slow improvement
Assini et al. [8]	60	M	20	Tetraplegia, areflexia, massive, dysautonomia (gastralgia, paralytic ileus, hypotension)	AMSAN	Normal	Not available	IVIGs rapid improvement.
Sedaghat et al. [9]	65	M	14	Tetra paresis, facial paresis, areflexia, hypoaesthesia, sensory deficit in lower limbs	AMSAN	Not available	Brain and cervical spine normal	IVIG, not available
El Otmani et al. [10]	70	F	03	Tetraplegia, areflexia, paranesthesia in all limbs, bilateral positive Lasegue sign	AMSAN	ACD	Not available	IVIG, not available

Many theories explain the mechanism of neurological manifestations of COVID-19. SARS-COV-2 receptors are expressed in the nervous system, making it a new neuropathogenic, and can also explain the symptoms of headache, weakness, neuropathies, altered sensorium, and stroke in patients of COVID-19 [11]. Annexation through the cribriform plate and olfactory bulb and dissemination through the trans-synaptic transfer are some of the other proposed mechanisms [12]. The medullary cardiorespiratory center invasion by SARS-CoV-2 is considered a factor for refractory respiratory failure observed in critical patients. However, the CSF findings in our case and multiple other cases showed normal white blood cell (WBC) count indicating the role of immune-mediated molecular mimicry in the causation of GBS, which is reported as a rare cause of GBS in COVID-19 patients.

Our case presented with progressive ascending paresis culminating in quadriplegia, areflexia, bilateral facial paresis, and sensory losses one week after having symptoms of fever, cough, and intermittent dyspnea. On admission, the patient was tested for COVID-19, and his RT-PCR came positive. The detailed neurological examination, electrophysiological studies, CSF, and MRI confirmed the diagnosis of GBS of the AMSAN variant. A course of IVIGs was given for five days, and significant improvement was seen.

## Conclusions

A similar pattern of presentation of GBS following the respiratory symptoms of COVID-19 in all the cases suggests it to be one of the complications of COVID-19. Therefore, a physician should keep this complication in mind while treating COVID-19 patients. There should be at least one follow-up visit of all the discharged COVID-19 patients. Keeping in view the course of the presentation of GBS, IVIGs should be started to prevent grave complications like respiratory paralysis, sepsis, pulmonary embolism, and cardiac arrest. However, whether GBS is caused by an immune response due to molecular mimicry or by direct neurological invasion in COVID-19 is yet to be confirmed and demands further clinical studies.
